# Nanoparticle–EMF synergism: a study on the combined effects on developmental and behavioral endpoints in *Drosophila melanogaster*

**DOI:** 10.3389/fpubh.2025.1645108

**Published:** 2025-09-16

**Authors:** Manisha Bhandari, Avnika Singh Anand, Kalyani Verma, Karuna Regmi, Amitabh, Dipti. N. Prasad, Ekta Kohli

**Affiliations:** Defense Institute of Physiology and Allied Sciences, New Delhi, India

**Keywords:** combinatorial effects, zinc oxide nanoparticle, electromagnetic field, *Drosophila melanogaster*, phenotypic abnormalities, longevity, multigenerational effects

## Abstract

The accelerated progression of modern technologies has exponentially amplified the pervasive presence of electromagnetic fields (EMFs) and nanoparticles (NPs) in various environments, from military arsenals to domestic settings. Despite extensive research on individual exposures, the cumulative effect of EMF and NPs co-exposure on biological systems remains poorly understood. This study investigates the combinatorial effects of 2.4 GHz EMF and zinc oxide (ZnO) NP exposure on *Drosophila melanogaster* across subsequent generations. We assessed various biological endpoints, including longevity, motor-neuronal responses, oxidative stress response, memory and learning responses, and phenotypic abnormalities. Flies were exposed to 2.4 GHz EMF for 10, 20, and 30 min independently and in combination with 0.1 mM and 0.5 mM ZnO NPs through the ingestion method. Our results showed that exposure to EMF significantly increased fly survival from day 14 to 50 following 10 min, with a more pronounced and sustained effect observed at 20 min (from day 14 to end of cycle). Independent exposure to 0.1 mM ZnO NPs had no observable effect on survival, whereas 0.5 mM NPs showed a steep decline from day 7. However, coexposure with 20-min EMF improved survival ability, inducing longevity from day 17 to 32 with 0.1 mM ZnO NPs, and from day 14 to 39 with 0.5 mM ZnO NPs. Behavioral impairments, elevated oxidative stress, and declined memory and learning abilities were observed. Furthermore, tergite patterning and pigmentation abnormalities were induced by EMF exposure, which were reversed over the subsequent generations. These findings highlight the complex, dose- and time-dependent biological responses to combined EMF and NP exposure. Our study emphasizes the need for further investigation into the potential risks and applications of these combinatorial interactions.

## Graphical abstract

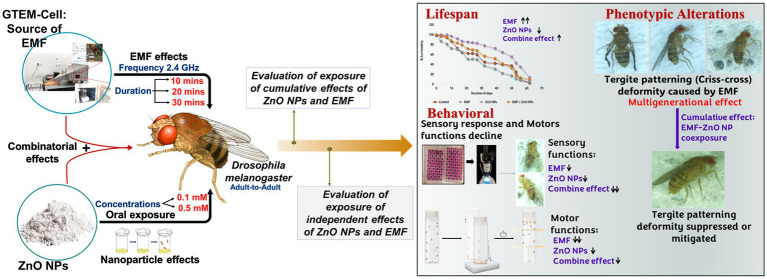

## 1 Introduction

Modern battlefield exposes soldiers to a unique combination of environmental stressors, including nanoparticles (NPs) and electromagnetic fields (EMFs), which evolve from the advanced photonic technologies, electronic devices, chemical warfare agents and explosions (1–5). However, beyond the battlefield, the presence of NPs and EMFs in everyday items such as household appliances, communication devices, cosmetics, and energy systems, as well as their roles in scientific research and medical applications, raises substantial concerns regarding their combined impact on biological systems (6–8).

EMFs, comprises of frequencies ranging from 3 Hz to 300 GHz which originates from electrical equipment, power lines, and wireless technologies (9). 2.4 GHz frequency is of particular interest because of its robust use in military sectors for surveillance, signal jamming, drone and robotic control, encrypted wireless data transfer, and secure short-range communications, and in civilian sector for Wi-Fi, Bluetooth devices, cordless phones, microwaves, and smart home gadgets. EMFs can have both thermal effects, characterized by an increase in tissue temperature (10), and nonthermal effects, including alterations in cellular signaling, gene expression, and oxidative stress, which are less understood and still controversial in the scientific community. Non thermal studies are emphasized to eliminate the potential perplexing influence of heat on biological processes.

Simultaneously, metal oxide (MO) NPs have complex interactions with biological systems; because of their small size, as these particles can easily enter cells and may potentially aid in drug delivery (11) and imaging (12). However, these particles can also pose risks, inducing oxidative stress and inflammation (10, 13). ZnO NPs are one of the most widely used MO NPs due to their unique physicochemical properties, antimicrobial activity, biocompatibility, photocatalytic behavior, cost-effectiveness, and wide applications in textiles, paints, sunscreens, food packaging, and biomedical devices (14–16). In the military sector, they are applied in antimicrobial wound dressings, UV-protective coatings, flame-retardant uniforms, and chemical-biological protective gear (17–20). Future work may also explore other NPs like TiO₂ and Ag for broader insights.

Previous studies have demonstrated that exposure to NPs and EMFs individually can lead to both therapeutic benefits (21, 22) and toxic effects (10, 23), like respiratory (24) and cardiovascular issues (25, 26) due to NPs, and neurological (27, 28), carcinogenic (29), reproductive (30, 31), and cognitive effects (32) caused by EMFs. Despite the abundance of data on individual exposure to NPs and EMFs, a significant knowledge gap remains regarding the biological effects of their combined interactions.

When NPs and EMFs coexist in an environment, they can undergo chemical and physical transformations due to their reciprocal mutual interactions. EMFs may exhibit unique behaviors, such as an enhanced absorption rate or scattering of electromagnetic waves, excitation of plasmonic resonance, hypothermic activation, and electroporation phenomena (33–36). Contrary, EMFs can affect NPs by changing their magnetic behavior (37), producing mechanical stress, causing charge accumulation or influencing their aggregation and dispersal (34), and causing localized heat or resonance phenomena (33). Such interactions may influence toxicity, therapeutic potential or biodistribution, and potential impacts on biological systems, yet this remains an underexplored area of research (38).

To address this knowledge gap, we used an *in vivo* model organism, *Drosophila melanogaster*. Commonly known as the fruit fly, serves as an essential model organism because of its relatively simple genome, which shares significant homology with that of humans, its short life cycle, and advanced genetic tools (39, 40). Its short generation time and high reproductive rate allowed us to assess multiple generations and study both hereditary and long-term biological impacts (39).

In this study, we investigated the impact of multigenerational non-thermal 2.4 GHz EMF and ZnO NP exposure on adult *Drosophila melanogaster* and their lifespan. Given the limited literature on their combinatorial effects on flies, this study offers novel insights into key biological functions with possible impacts on health and ecological systems. By simulating realistic coexposure conditions, this work aims to better understand the potential health risks associated with the coexistence of EMFs and NPs in both military and civilian environments.

## 2 Materials and methods

### 2.1 *Drosophila* strain and culture conditions

The Oregon R strain of *Drosophila melanogaster* was selected as the model organism for our *in vivo* experimental studies. Fly cultures were maintained in a controlled environment (incubator) at 25 ± 1 °C with a relative humidity of 60 ± 5% and a 12:12 h light–dark cycle. The flies were reared on a standardized diet consisting of agar, cornmeal, dextrose anhydrous, sucrose and yeast extract [Cat No. 1D001-1 L, Himedia Pt. Ltd., India] with the addition of 80% propionic acid or nipagin (methyl-p-hydroxy benzoate) to prevent fungal contamination. These optimized conditions ensured optimal growth, reproduction, and experimental reproducibility. Both the control and treated groups were maintained under identical conditions throughout the study to minimize environmental variables and ensure reliable results.

### 2.2 Exposure

**Figure 1:**
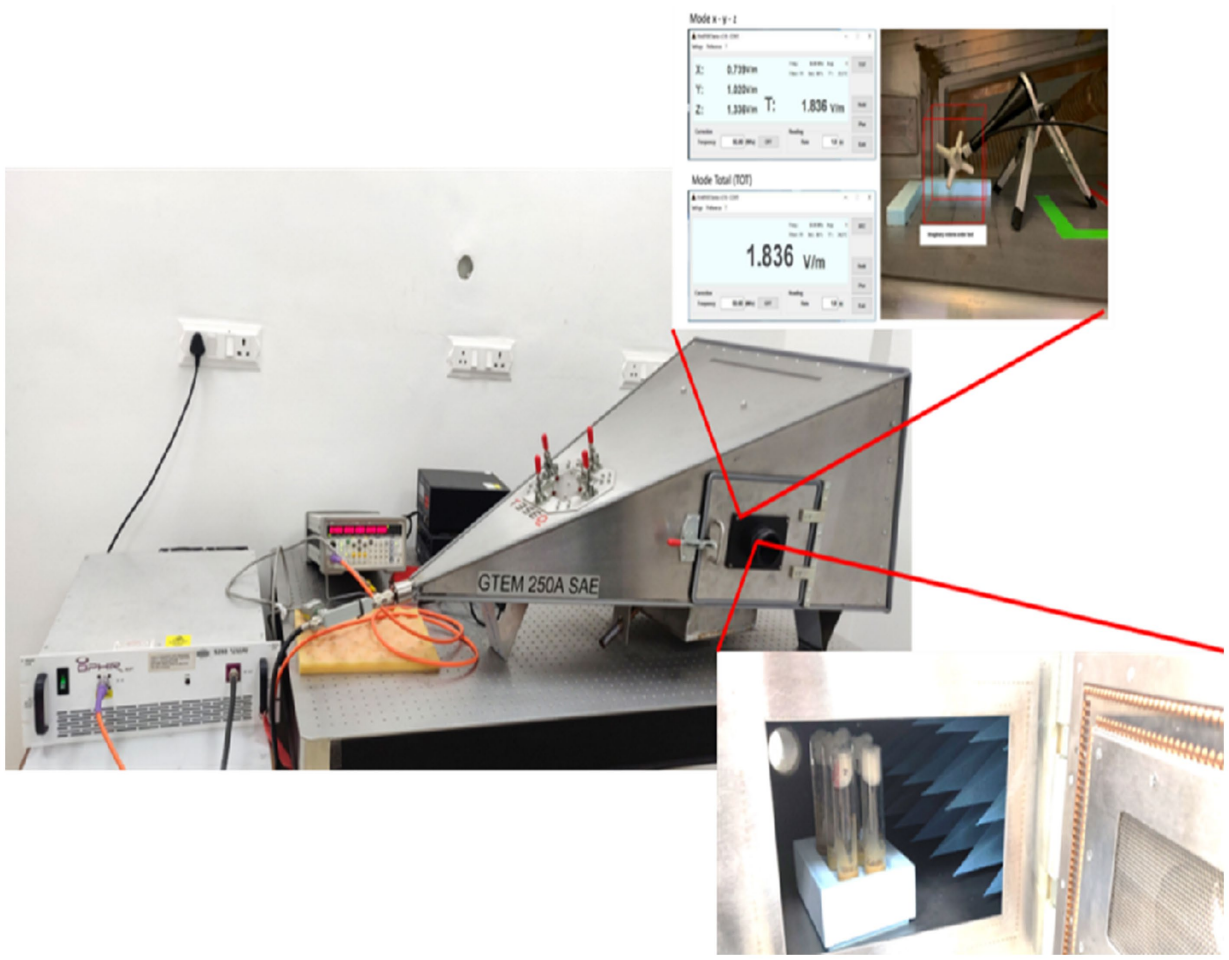
Image of the horn-shaped GTEM cell (GTEM 250A SAE model) used for EMF exposure.

EMF exposure was performed via a gigahertz transverse electromagnetic (GTEM) cell [GTEM 250A SAE model]. The EMF source was a certified horn-shaped GTEM cell (Figure 1) with a power density of 916 mW, an electric field strength of 44.29 V/m, and an amplitude of −20 dBm. The Oregon R strain of *Drosophila melanogaster* was exposed to 2.4 GHz HF-EMFs for durations of 10, 20, and 30 min. The experimental groups of *Drosophila* were meticulously organized based on two key variables: the dosage of ZnO NPs and the duration of exposure to EMF.

ZnO NPs [CAS No. 1314-13-2, >97% Catalog No. 677450-5G, Sigma Chemical Co. Ltd., USA] were used for this study because of their extensive application in various fields. ZnO NPs were spherical with a size of 23 ± 2 nm (Transmission Electron Microscopy) and the mean hydrodynamic size of particle was 110 ± 2 nm in Dulbecco’s Modified Eagle Medium (Dynamic Light Scattering), with a polydispersity index of 0.2 (41). The flies were exposed to ZnO NPs via the ingestion method. ZnO NPs were weighed and dissolved in Milli-Q water to achieve concentrations of 0.1 mM and 0.5 mM, selected based on existing literature and prior lab studies demonstrating their biological relevance, while representing sub-toxic to moderately toxic levels commonly used in nanotoxicology research (41, 42). The solutions were vortexed for homogeneity and sonicated for 30 min at 25 °C, followed by centrifugation at 13,400 rpm for 30 min. The supernatant was collected and then mixed with the *Drosophila* diet.

*Drosophila melanogaster* adult flies were exposed to high-frequency EMFs with or without a ZnO NP-containing diet. Eight- to ten-day-old adult flies (10 males and 10 females) were randomly selected and added to each tube on the day of exposure. All the groups were organized in triplicate, and the experiments were carried out three times to ensure significance.

To investigate the combined effects of ZnO NPs and EMF radiation on flies, a total of 12 groups of fly vials (*n* = 20; 10 males and 10 females per vial) were used. The experimental design consisted of three main groups. Group 1 was tested for only EMF exposure, in which the flies were exposed to EMF alone for 10 min, 20 min and 30 min for 7 days. Group 2, in which the flies were exposed to ZnO NPs at two different concentrations, 0.1 mM and 0.5 mM, without EMF interference. The third group (Group 3) was exposed to the combinations of NPs and EMF, this group was further divided into three subgroups with durations of 10 min, 20 min, and 30 min were used for each concentration (0.1 mM and 0.5 mM). The control groups were kept in separate vials under the same conditions, and all the experimental vials were evaluated under blinded conditions to ensure unbiased results.

### 2.3 Longevity

To study the effects of ZnO NPs and EMF on lifespan, we employed a longevity assay in *Drosophila*. Flies were maintained in their standard environment. Twenty newly emerged flies (10 male and 10 female) were reared on diets with or without NPs and further divided into two groups: one with only EMF exposure and the other without EMF exposure. To maintain consistent feeding conditions and prevent larval interference, the flies were transferred to fresh vials every fourth day. Throughout the experiment, the number of dead and live flies was carefully recorded at each transfer. The experiment was conducted for a duration of 60 days. Survival curve was generated from the recorded data.

### 2.4 Crawling assay

The crawling assay was used as a behavioral and neurological assessment tool to evaluate larval locomotion, leveraging the well-established correlation between crawling patterns and neuronal function. To facilitate this analysis, a 1.5% agar substrate was prepared in a petri dish, which provided a controlled environment for larval movement. Specifically, third instar larvae were collected for this study, and then the larvae were rinsed with MilliQ to eliminate residual food debris. The larvae were subsequently placed on the agar for 30 s, after which it was allowed to leave distinct trail marks indicative of their locomotor activity. This methodology enables the quantitative analysis of larval crawling behavior by measuring the distance travelled by the larvae (Supplementary Figures S1, S2). This assay was conducted with triple replicates to enable comparisons of crawling behavior across various experimental conditions. The distance travelled by the larvae over 30 s was quantified via a graph paper grid with 0.1 cm^2^ squares, and the data analysis was performed via ImageJ software.

### 2.5 DCFDA-ROS measurement

The DCFDA assay was used to measure intracellular ROS and evaluate oxidative stress induced by combined exposure to ZnO NPs and EMF in *Drosophila melanogaster*. The assay utilized the cell-permeable dye 2,7-dichlorofluorescein diacetate (DCF-DA), which is converted to fluorescent 2,7-dichlorofluorescein upon reacting with hydrogen peroxide. The 2,7-dichlorofluorescein emitted fluorescence detectable by spectroscopy at excitation and emission wavelengths of 495 nm and 529 nm, respectively (43). The third-instar larvae were collected, washed three times with Milli-Q water to remove food residues, and then homogenized in ice-cold 1 X PBS. The homogenate was subsequently centrifuged at 5,600 × g for 10 min, after which the supernatant was treated with 15 μM DCF-DA at an equal ratio. Following a 20-min incubation at 37°C, the fluorescence was measured with excitation at 485 nm and emission at 520 nm. The results are presented as the relative fluorescence intensity normalized to that of the control larvae.

### 2.6 Two-choice taste assay

*Drosophila melanogaster* gustatory preferences and taste perception were investigated using a two-choice taste testing method. For this method, the flies were starved for overnight before being put in a 96-well plate (a taste test chamber) with agar gels that contained either water or sucrose (red dye, acid fuchsin). After a two-hour feeding period, the flies were anaesthetized on ice, and their abdomens were examined under a stereomicroscope for appearance of color, which were then classified as red, blue, mixed, or uncolored.

### 2.7 Phenotyping

In this study, flies from two subsequent generations (F1 and F2) were comprehensively examined under a stereo microscope (OLYMPUS Co. Japan) for phenotypic abnormalities caused by NPs and EMF exposure. Following exposure, the wings, eyes, thorax, body color, and other physical features were examined for potential abnormalities. Each treatment was conducted twice to ensure accurate and consistent outcomes. Since the exposure was repeated three times, approximately 300 and 450 flies were screened for phenotypic abnormalities for each treatment, for a total of 1,000 to 1,300 flies per generation.

### 2.8 Statistical analysis

Statistical analysis of the data was performed via GraphPad Prism software (version 5). All the data are presented as the means ± standard deviations. The analysis included the application of Bonferroni’s multiple comparison test, and the calculation of the arithmetic mean with standard deviation via one-way ANOVA. Two-way ANOVA was used to analyse the survival results. A *p* value of ≤ 0.05 was used to define significance for all tests.

## 3 Results

### 3.1 Longevity

**Figure 2:**
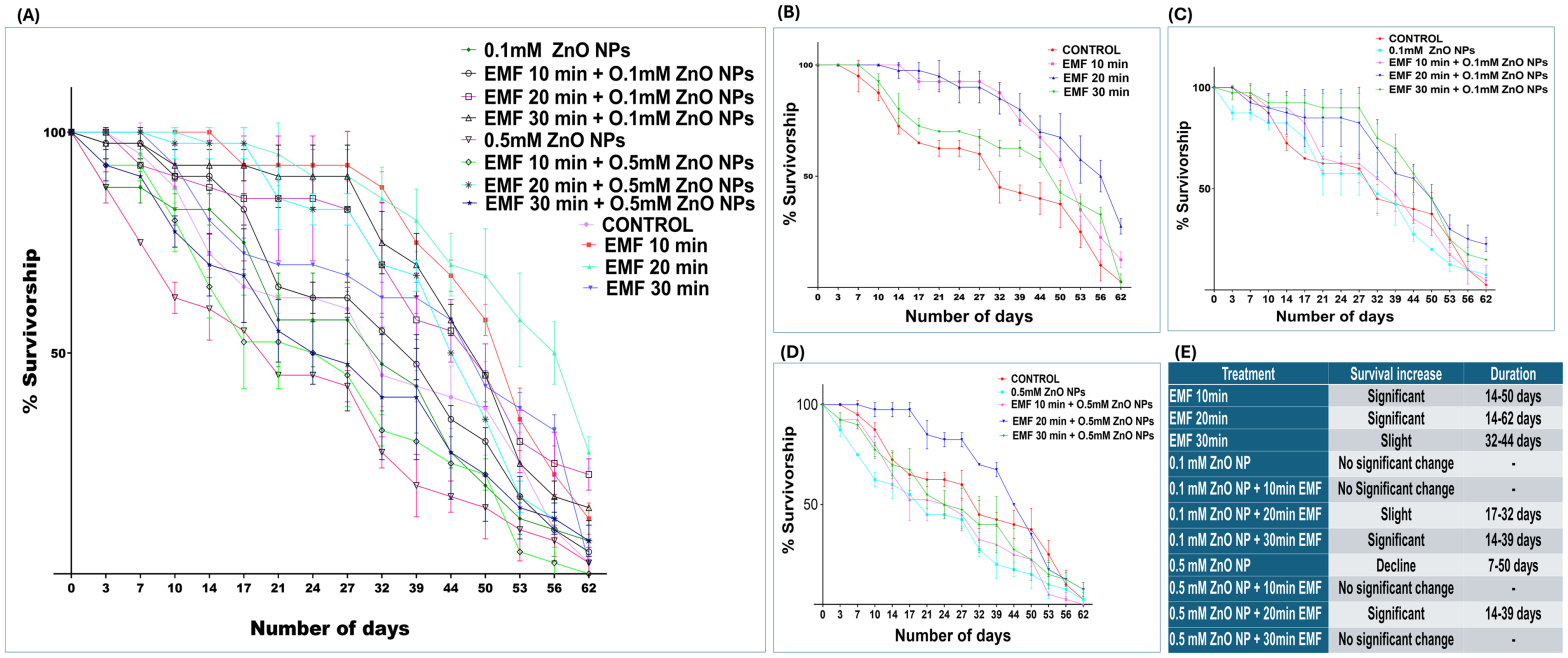
Percent survival of adult flies exposed to EMF, ZnO NPs, and co-exposure. **(A)** Survival rates under EMF, ZnO NP, and combined exposure conditions vs. the control. **(B)** Percent survival under EMF exposure vs. the control. **(C)** Percent survival with 0.1 mM ZnO NPs vs. co-exposure to EMF. **(D)** Percent survival with 0.5 mM ZnO NPs vs. co-exposure to EMF. **(E)** Table representing significant days. The data are represented as the means ± standard deviations, with error bars indicating variability.

Survivalship assay, performed on flies exposed to EMF and ZnO NPs independently and in combination between duration of EMF exposure and concentrations of the NPs showed subtle interaction. When flies were exposed solely to EMF, there was an observable, time-dependent increase in survival, as shown in Figure 2B. The survival rate of flies exposed to EMF for 10 min increased significantly from day 14 to day 50 (Figure 2E). This trend was intensified with 20 min of EMF exposure, leading to a prolonged increase in lifespan that lasted from day 14 till end-of-life cycles. A slight but noteworthy increase in survival was also observed in flies exposed to 30 min of EMF, but the effect was more transient, lasting from 32 to 44 days.

Flies exposed to 0.1 mM ZnO NPs alone did not exhibit significant changes in survival (Figure 2A). However, when EMF was combined with 0.1 mM ZnO NPs, the longevity of the flies clearly improved (Figure 2B). Specifically, 20 min of EMF exposure slightly increased survival at 17 and 32 days. A more pronounced survival benefit was observed when EMF exposure was extended to 30 min in combination with 0.1 mM ZnO NPs, with a significant increase in longevity from 14 to 39 days (Figure 2E).

Flies exhibited a marked decline in survival when exposed to 0.5 mM ZnO NPs alone, with survival decreasing steadily from day 7 to day 50 (Figure 2E), indicating toxic effects. However, when EMF was combined with 0.5 mM ZnO NPs, the toxic effects caused by ZnO NPs were suppressed or mitigated to some extent. When combined with 20 min of EMF, 0.5 mM ZnO NPs significantly improved longevity at 14 to 39 days. The 0.5 mM ZnO NPs and EMF treatment for 10 min resulted in a steep decline in survival, which was recorded around day 53. These results imply that the harmful effects of 0.5 mM ZnO NPs might be countered by the protective effects of EMF at 20 min. In conclusion, EMF exposure, particularly at a duration of 20 min, positively influences the longevity of flies, both independently and in combination with ZnO NPs (Figure 2A).

### 3.2 Crawling

**Figure 3:**
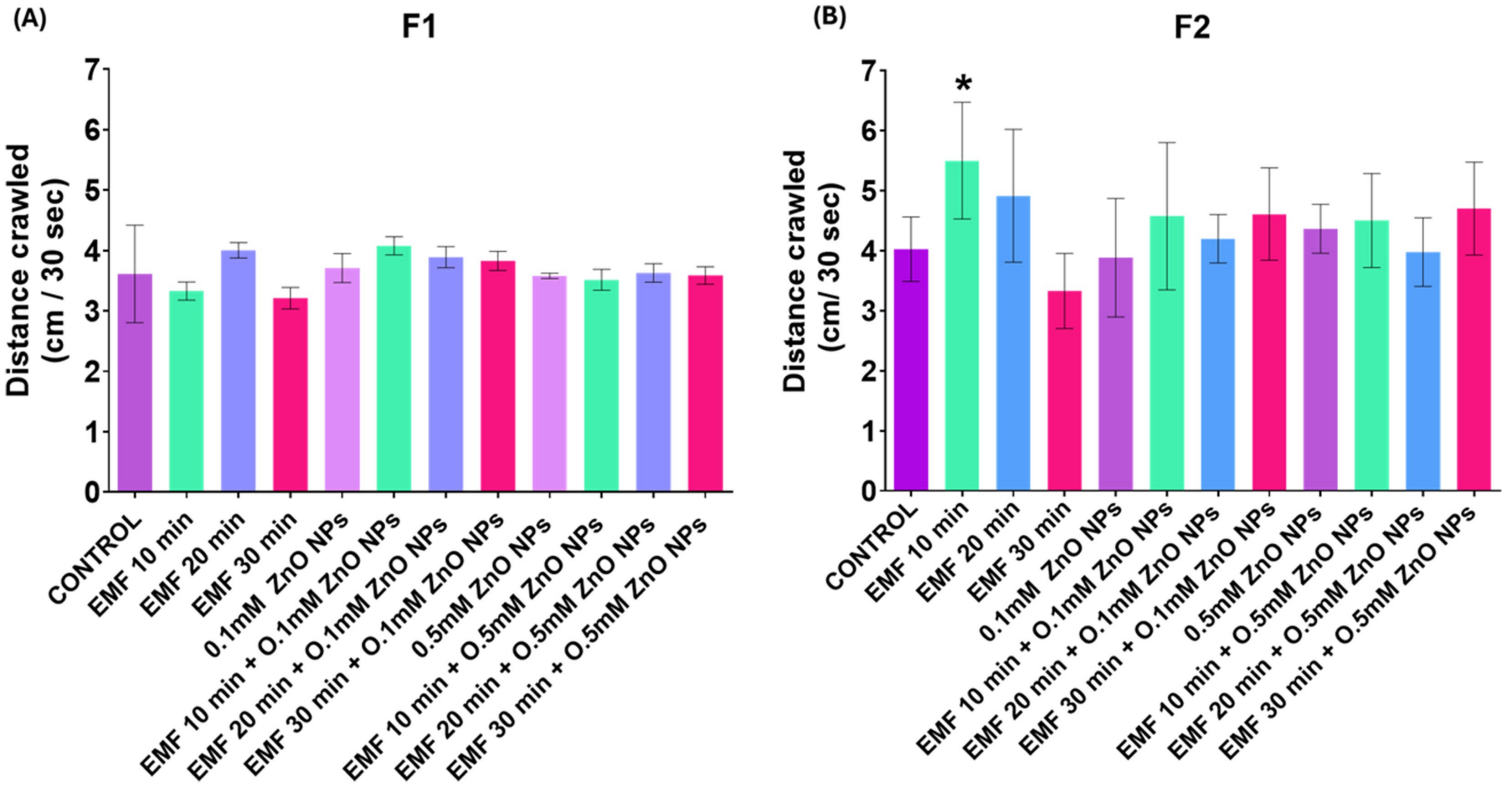
Crawling behavior of third instar larvae exposed to EMF (10, 20, and 30 min), ZnO NPs (0.1 mM and 0.5 mM), and their co-exposure. The bar graph presents a comparative analysis of the various exposure conditions in the **(A)** F1 and **(B)** F2 generations. The bars indicate the mean values, whereas the error bars represent the standard error of the mean. The bar graph shows the means ± standard deviations, and statistical significance is denoted by *** (*p* < 0.01).

In the F1 progeny, 30 min of EMF exposure caused a slight decline in crawling behavior (Figure 3A), suggesting a potential inhibitory effect on motor activity or neuronal function. However, in the F2 progeny, EMF exposure for 10 min enhanced the crawling ability of the larvae (Figure 3B). When the flies were exposed to the combination of EMF and ZnO NPs, no significant alterations in crawling behavior were detected, indicating that the interaction between EMF and the NPs does not substantially affect motor function.

### 3.3 Oxidative stress

**Figure 4:**
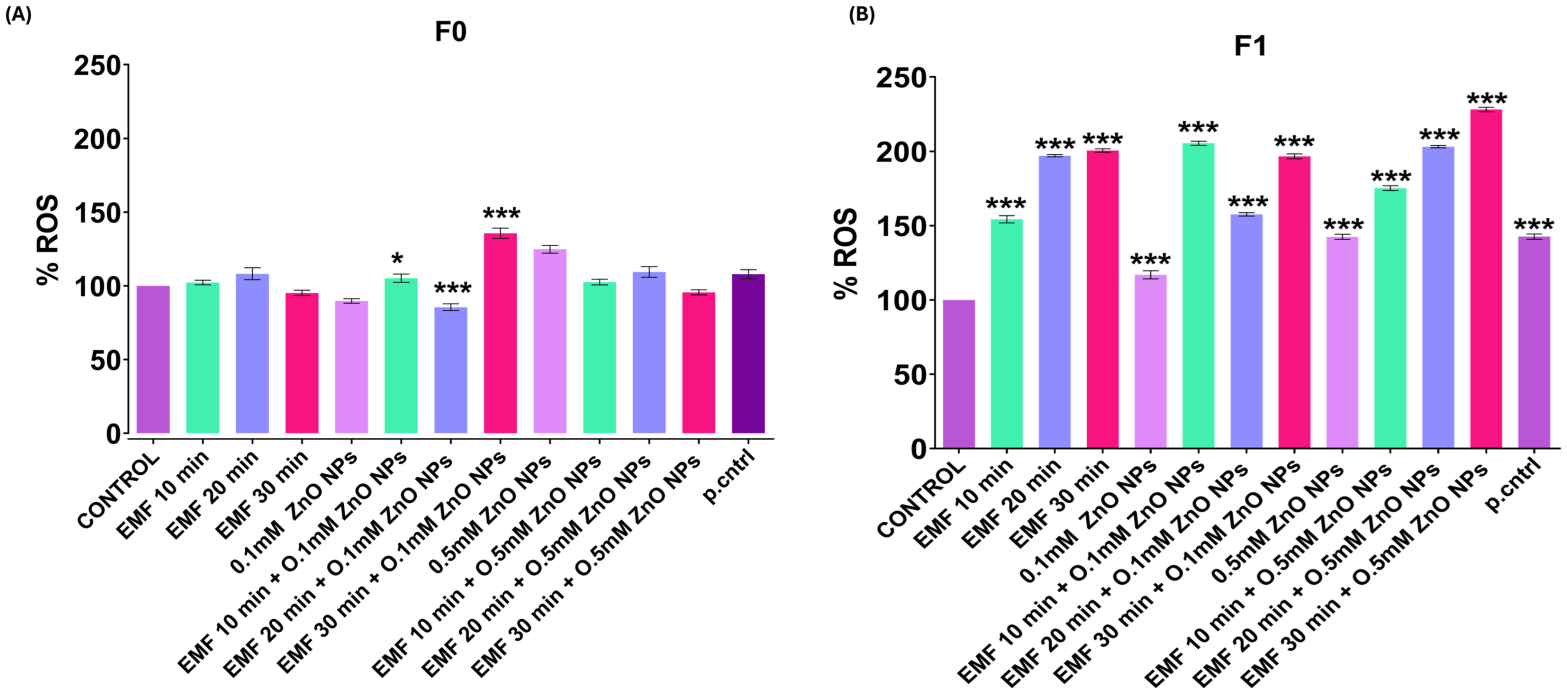
ROS levels in adult flies following EMF and ZnO NP exposure quantified using DCFDA assay and expressed as relative fluorescence intensity units per mg of protein (control = 100%). The bar graph shows ROS levels in **(A)** F0 (parent flies) and **(B)** F1. The data are presented as means ± standard deviations, and statistical significance is indicated by *** (*p* < 0.01).

In the parent flies (F0), no significant changes in oxidative stress levels were observed, neither when exposed to ZnO NPs and EMF individually nor when exposed to their combination. Only a significant induction of oxidative stress was noted when the flies were exposed to a combination of 0.1 mM ZnO NPs and EMF for 20 and 30 min (Figure 4A). Additionally, exposure to 0.5 mM ZnO NPs alone resulted in a significant increase in reactive oxygen species (ROS). Although there were no notable alterations in the parent generation, the flies in the F1 generation showed increased levels of oxidative stress, suggesting a multigenerational effect from parental exposure (Figure 4B). Furthermore, ROS significantly increased with exposure to 0.5 mM ZnO NPs alone.

### 3.4 Two choice tasting assay

**Figure 5:**
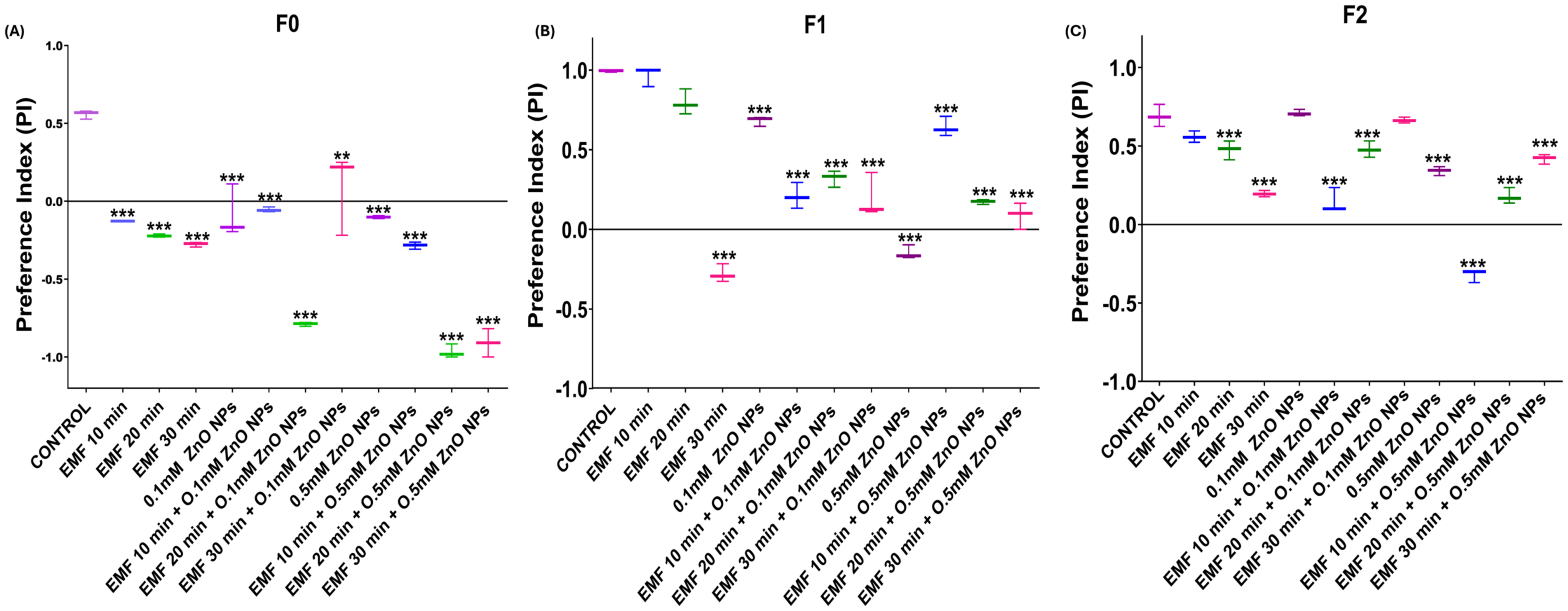
Two-choice feeding preference assay in adult flies exposed to EMF and ZnO NPs. PIs of flies in **(A)** F0 (parent), **(B)** F1, and **(C)** F2 progeny. The data are presented as means ± standard deviations, with statistical significance denoted by *** (*p* < 0.01).

The two-choice tasting assay was used to assess the effect on sensory abilities caused by co-exposure to ZnO NPs and EMF. The preference index (PI) of parent flies (F0) subjected to EMF for 10, 20, and 30 min showed a gradual deterioration, with the most notable damage occurring at 30 min (Figure 5A). However, in F1 progeny, a significant decline in the PI was observed at 30 min of exposure, with no statistically significant effects at 10 and 20 min, indicating a reduced but still noticeable transmission of the deficit (Figure 5B). In F2 progeny flies, a significant decline was observed at both 20 and 30 min of EMF exposure (Figure 5C), demonstrating a multigenerational effect.

Compared with the individual effects of EMF, the co-exposure of parent flies (F0) to 0.1 mM ZnO NPs and EMF led to a more pronounced decline in PI; however, a marked decline was observed at 10 and 20 min, followed by partial recovery at 30 min, suggesting fluctuating sensitivity across generations (Figure 5B). The negative impact on F1 generation flies was less pronounced than that on F0 generation flies, while F2 progeny flies regained the same results as parent flies (Figure 5C).

In the F0 generation, a significant decline in the PI was observed with 0.5 mM ZnO NP exposure alone, which was further exacerbated when ZnO NPs was combined with EMF, particularly at 20 and 30 min (Figure 5A). In the F1 generation, the combined EMF and ZnO NP treatment also led to significant decline across all exposure durations in comparison with that of the control. However, relative to ZnO NP exposure alone, there was a slight improvement in sensory function under the combined treatment, indicating that F1 flies may exhibit a minor adaptive response that either suppresses or mitigates the effects of ZnO NPs under EMF stress. In the F2 generation, the combine treatment caused a significant decrease in taste perception at 10 and 20 min, with some improvement observed at 30 min compared with that observed after ZnO NP exposure (Figure 5B).

### 3.5 Phenotyping

**Figure 6:**
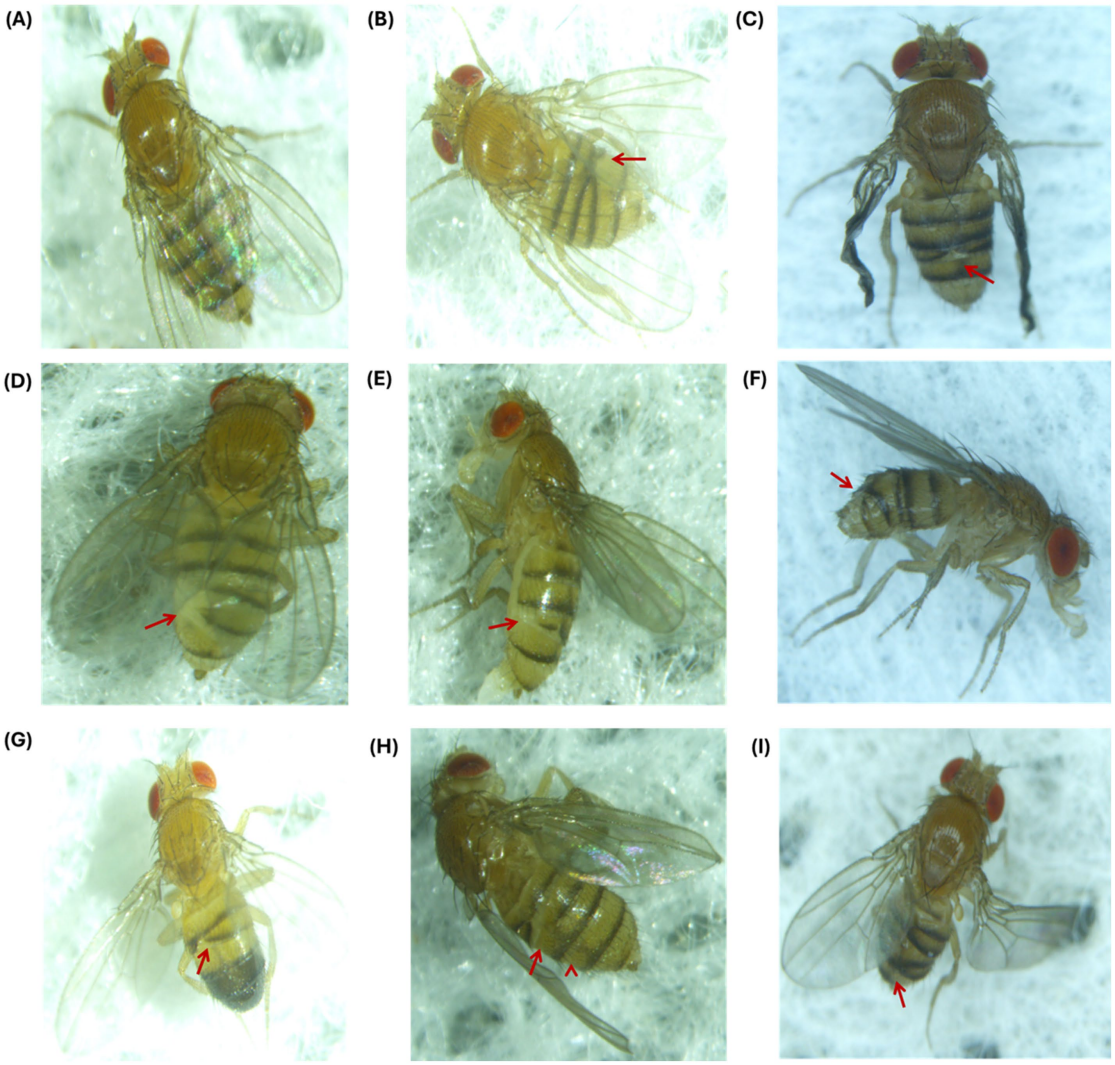
Phenotypic abnormalities in F1 and F2 progeny after EMF exposure for 10, 20 and 30 min. **(A)** Control fly with a normal phenotype. At 10 min **(B)** F1 flies exhibited a crisscross pattern of tergites, and **(C)** presented crumpled wings and a crisscross pattern of tergites in the F2 generation. At 20 min **(D, E)** tergite deformation and loss of cuticle pigmentation occurred in F1, and **(F)** a crisscross-tergite pattern was observed in F2. At 30 min, criss-cross tergite pattern was observed in **(G)** F1 males, **(H)** F1 females, and **(I)** F2 flies.

To study the effects of EMF, ZnO NPs, and their co-exposure, flies were carefully examined under a stereomicroscope for visible morphological changes in *Drosophila*. Flies were screened for subsequent generations: F0 (parent flies), F1, and F2. Phenotypic analysis revealed that exposure to EMF at a frequency of 2.4 GHz led to visible morphological changes in the flies, specifically the crisscross pattern of tergites (Figure 6) and the lack of pigmentation at tergites. These aberrant phenotypes were heritable and passed on to the next generation, indicating that EMF exposure alone induced heritable phenotypic alterations in tergite patterning. When exposed to a combination of 30 min of EMF exposure and 0.1 mM ZnO NPs, F2 progeny flies presented pigmentation deformities in tergites (Supplementary Figure S3).

## 4 Discussion

Simultaneous exposure to EMFs and NPs is becoming more common as technology continues to advance and become more integrated into our daily lives. The combined exposure of EMFs and ZnO NPs yields an interaction that can modulate multiple outcomes, either increasing toxicity, suppressing it or eliminating completely. It is essential to understand these combined effects to accurately assess any potential risks to the environment and human health. In pursuit of this understanding, this study investigated the combined effects of EMF and ZnO NPs on biological functions of *Drosophila melanogaster*, focusing on biological endpoints including lifespan, motor function, oxidative stress, sensory impairments, and heritable phenotypic alterations.

One key finding of our study was the significant impact of EMF exposure on lifespan (Figure 2), particularly when EMF was combined with 0.1 mM of ZnO NPs. Exposure to EMF alone increased survival in a time-dependent manner, particularly at shorter periods of exposure for 10 and 20 min. These results align with previous reports suggesting that low-level EMF exposure can induce protective cellular responses via mild oxidative stress, a concept known as hormesis (44, 45). The idea of hormesis (45, 46), in which low-dose environmental stresses initiate adaptive cellular pathways that enhance resilience and longevity (47, 62, 63). Our findings suggest that EMF exposure can independently increase lifespan and also influence NP toxicity, depending on the concentration. Low ZnO NP concentrations (0.1 mM) when combined with low-level EMF exposure durations (10 and 20 min) considerably increased lifespan. ZnO NPs in higher concentrations (0.5 mM) reduced lifespan, likely due to cellular dysfunction and oxidative damage. This aligns with existing literature which has demonstrated that NPs can interact with biomolecules and penetrate cell membranes, disrupting cellular processes or damaging proteins (48–50). According to our study, 20 min of EMF exposure improved the toxic effects caused by relatively high dose of ZnO NPs (0.5 mM). These findings suggest that EMF exposure can influence stress–response pathways, potentially offering a protective advantage when combined with lower concentrations of NP (0.1 mM). We observed a time-dependent improvement in survival associated with EMF exposure, particularly at shorter periods (10–20 min), which is contrary with previous research that indicate detrimental effects of EMF exposure (51, 52). This disparity emphasizes that further investigation is required to completely understand the multifaceted impacts of EMFs. To the best of our knowledge this study is the first to disclose the combine effects of ZnO NP and EMF exposure on *Drosophila* lifespan, as there is not any direct comparable evidence currently. Nonetheless, our findings lay the groundwork for more research into this interaction and its implications on longevity and cellular stress responses.

Further in support of transgenerational impact, we observed that the ROS level was minimal in parent flies but elevated in the progeny. These findings are consistent with prior research by (53), who observed that prolonged environmental stress could trigger heritable oxidative stress responses in *Drosophila.* These heritable effects may be explained by epigenetic changes, like modification in DNA packaging or the activity of non-coding RNAs, which persist across generations even after the original stress is removed. Similarly, the decline in motor function observed in the crawling assays supports existing evidence that NPs and EMFs independently impair neuromuscular function (53, 54), however, our study also observed that their combination intensified the behavioral damage, particularly in offspring.

Co-exposure of EMFs and ZnO NPs resulted in a significant reduction in taste perception in the directly exposed generation (F0), indicating that the nervous system is especially sensitive to these stressors. Similar findings were reported by other studies that highlighted how combined environmental stressors such as heavy metals and NPs caused cumulative damage to sensory and cognitive functions in animal models (55). The sensory deficits in this study also mirror outcomes in other model organisms where EMF exposure led to impaired sensory perception and cognitive deficits (10). These alterations were more prominent in the F1 and F2 generations, implying that cumulative exposure and multigenerational transmission play an important role in changing sensory function over time.

To solidify the basis of our study, phenotypic analysis also provided additional evidence of heritable effects and mitigating effects of EMF exposure and the combination of EMF and ZnO NPs. In our study, we observed a heritable tergite patterning defects such as crisscross pattern in the tergites of exposed flies. The unusual crisscross arrangement of bristles and cuticular structures, notably on the dorsal segments (tergites), indicates an underlying disturbance in the signaling pathways that determine cell polarity and segmental identity. The *hedgehog (hh)* and *engrailed (en)* signaling axes are two important routes involved in the creation of tergite patterns. *hh* functions as a morphogen, influencing cell polarity in neighboring segments (56–58). Previous study has shown that alterations in *hh* signaling cause altered cell fates and segmental mis patterning (59). Our findings support Meinhardt’s 1983 (60) morphogen gradient hypothesis, which claims that environmental disruptions, such as NP and EMF exposure, may influence with the diffusion of morphogen (e.g., hedgehog), resulting in misinterpretation of positional cues and aberrant patterning. The absence of conventional intersegmental borders may further contribute to the crisscross patterning.

Our findings also correspond to the literature on pigmentation variation, specifically the significance of the ebony gene in tergite pigmentation. According to the existing literature ebony is essential for melanin biosynthesis pathway and is responsible for the clinical heterogeneity in tergite pigmentation found across different *Drosophila* populations (61). Mutations in ebony’s cis-regulatory regions alter not only pigmentation intensity but also a variety of behaviors such as circadian rhythms and visual function. Our study indicates that the combination of EMF exposure and ZnO NPs can mitigate these disruptions, favoring more normal pattern formation by strengthening protective cellular mechanisms. This could pave the way for future research into how the environment modulates gene signaling throughout development. However, the specific mechanisms supporting our study have yet to be thoroughly explored.

The findings of the present study suggest that EMF exposure can independently increase survival and improved the toxicity induced by ZnO NPs. Combined exposure to EMFs and ZnO NPs intensifies oxidative stress, motor function impairment and sensory deficits, which deteriorate progressively across subsequent generations. EMF exposure induces heritable phenotypic deformities, and co-exposure reversed these phenotype deformities.

## 5 Conclusion

This study reveals that coexposure to ZnO NPs and EMF in *Drosophila melanogaster* induces complex, dose dependent biological responses including altered lifespan, oxidative stress, motor function, sensory perception, and heritable phenotypic changes. EMF exposure alone, at varied durations, enhanced longevity and induced heritable phenotypic alterations, while combined exposure with ZnO NPs showed either protective or adverse outcomes, depending on exposure conditions. Our findings suggest that the interplay between NPs and EMFs is complex, likely involving epigenetic or adaptive cellular responses, and may pose long-term biological risks. The findings, whether referring to NPs or EMFs, highlight the importance of prioritizing safety concerns as exposure to these technologies’ increases, possibly jeopardizing future generations as well as the current generation. To enable their safe and responsible deployment, scientific research and regulatory frameworks should prioritize methodically analysing and mitigating potential risks as these industries evolve. Further research is needed to understand the underlying mechanisms and assess the long-term ecological and health implications of combined EMF and NP exposure.

## Data Availability

The raw data supporting the conclusions of this article will be made available by the authors, without undue reservation.
