# Supplementary material for: Nanoparticle–EMF synergism: a study on the combined effects on developmental and behavioral endpoints in Drosophila melanogaster
Source: Front Public Health. 2025 Sep 16;13:1645108. doi: 10.3389/fpubh.2025.1645108 (PMC12479256; doi:10.3389/fpubh.2025.1645108)
Supplement: Supplementary file 1 [file Table_1.DOCX]

**SUPPLEMENTARY INFORMATION**

**Nanoparticle-EMF Synergism: A study on the combined effects on Developmental and Behavioral Endpoints in *Drosophila melanogaster.***

Manisha Bhandari*, Avnika Singh Anand*, Kalyani Verma, Karuna Regmi, Amitabh, Dipti. N. Prasad, Ekta Kohli***

Defense Institute of Physiology and Allied Sciences, Timarpur, Delhi, India.

*****Corresponding author:**

Dr. Ekta Kohli,

Scientist ‘F’, Defense Institute of Physiology and Allied Sciences, DRDO

Lucknow Road, Timarpur, Delhi-110054, India

Tel: 011-23905238

Email: [ektakohli@hotmail.com](mailto:ektakohli@hotmail.com)

* These authors share first authorship


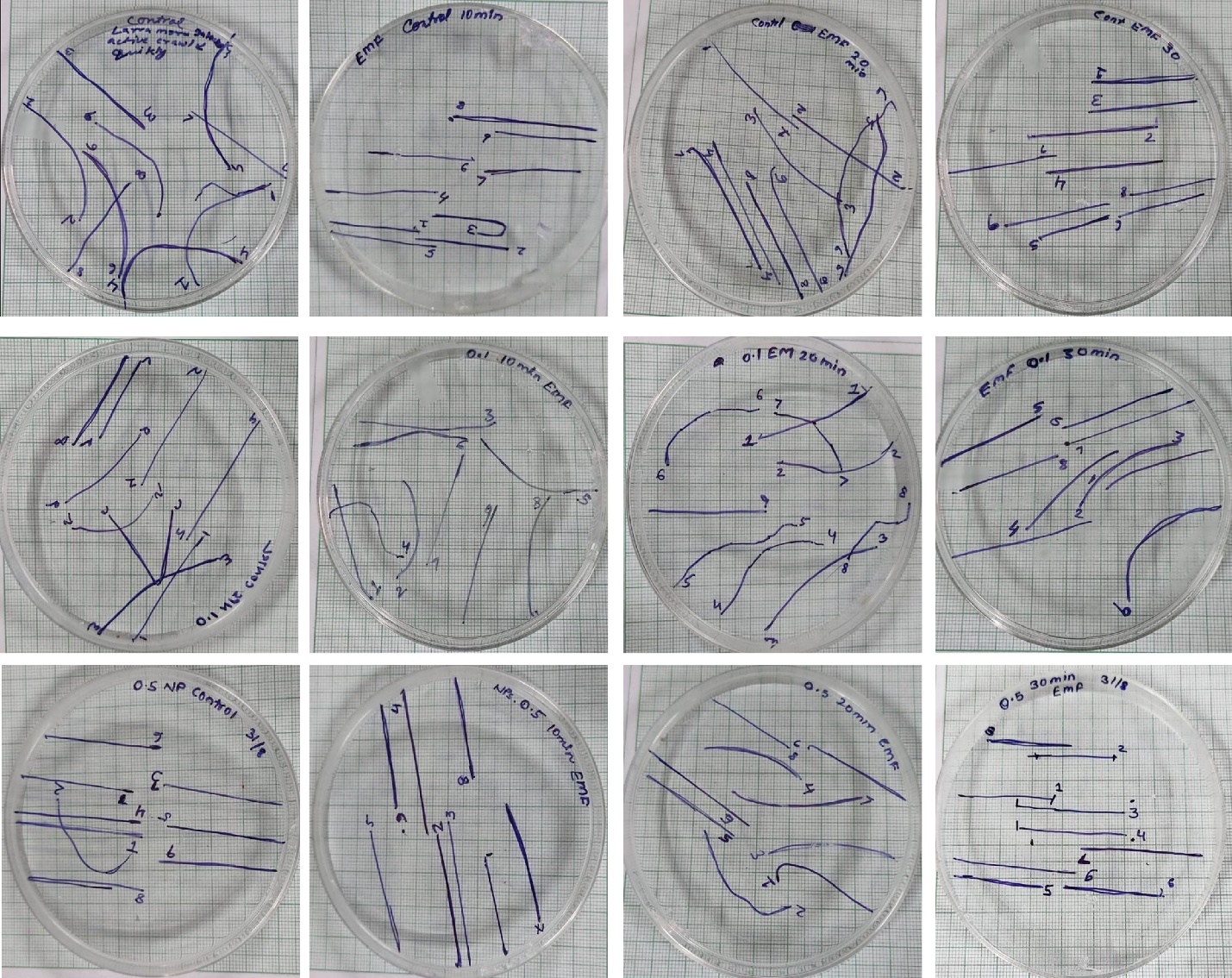


**Figure S1**: Image showing agar plates with path trailed marks of distance covered by treated third instar larvae in F1 generation.


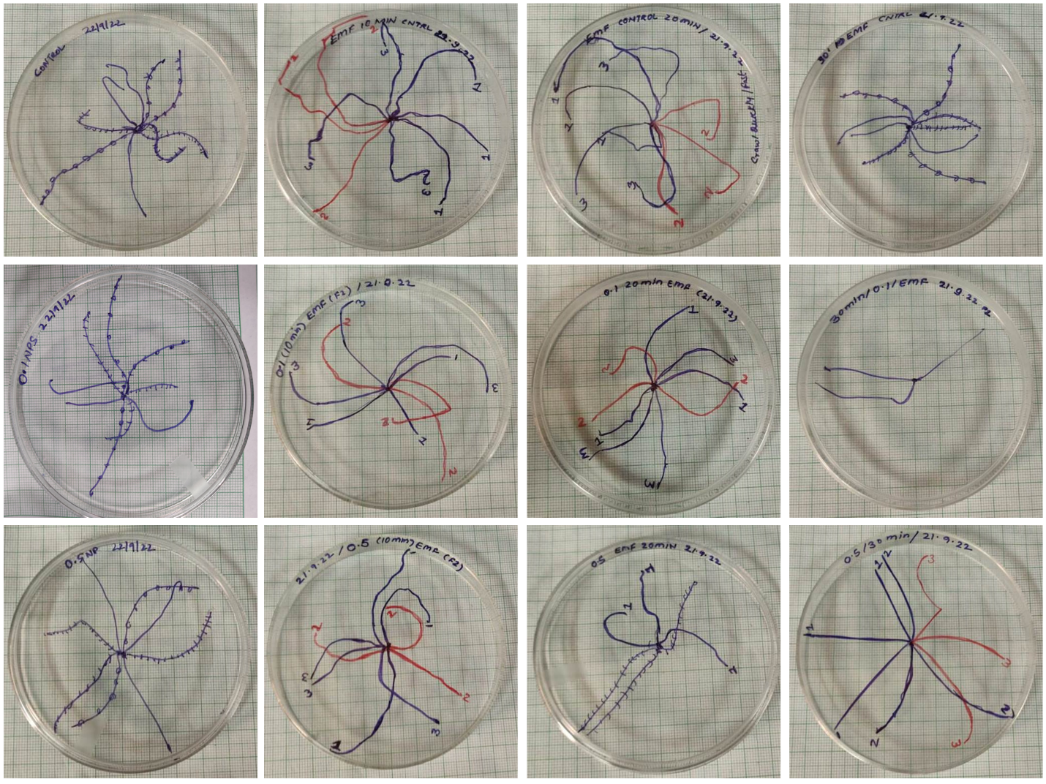


**Figure S2**: Image showing agar plates with path trailed marks of distance covered by treated third instar larvae in F2 generation.


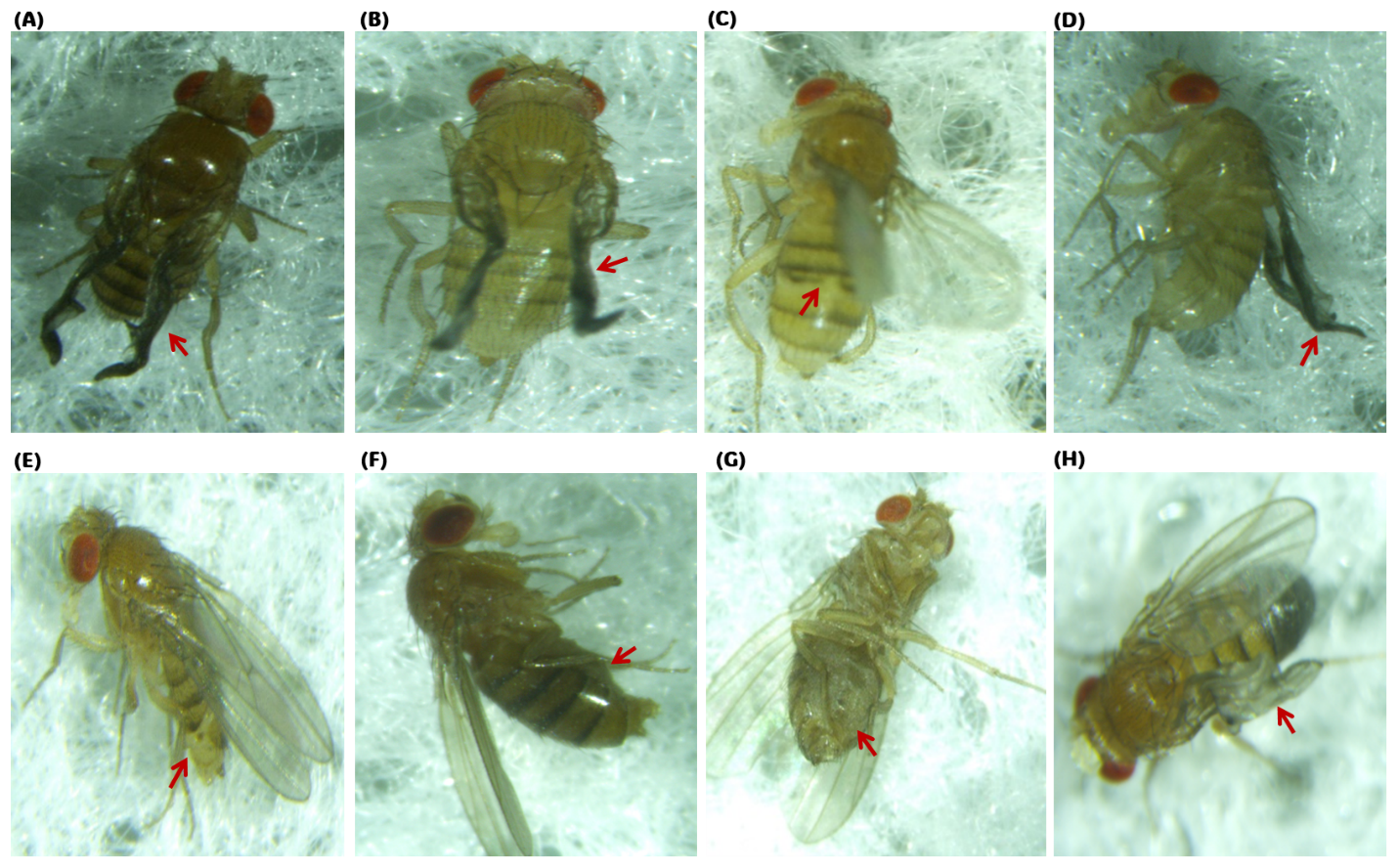


**Figure S3**: Images showing phenotypic abnormalities when exposed to (A, B) ZnO 0.1mM + EMF 10 min F1, wrinkled and curly wings; (C) ZnO 0.1mM + EMF 30 min F2, missing tergite; (D) ZnO 0.1mM + EMF 30 min F1, wrinkled and curly wings; (E) ZnO 0.5mM + EMF 20 min F1, tergite pattern disruption; ZnO 0.5mM (F, G) body pigmentation and, (H) wing damage.
